# The Development and Clinical Impact of an Innovative Palliative Care Lever Tool for Individuals With Idiopathic Pulmonary Fibrosis: A Quality Improvement Project

**DOI:** 10.1177/10499091241304443

**Published:** 2024-11-29

**Authors:** Kathryn Fenwick, Maryl Kreider, Jeannette Kates

**Affiliations:** 1 Penn Interstitial Lung Disease and Sarcoidosis Program, Harron Lung Center, Perelman Center for Advanced Medicine, 6569 Penn Medicine, Philadelphia, PA, USA; 2 School of Nursing, 6572 University of Pennsylvania, Philadelphia, PA, USA

**Keywords:** palliative care, idiopathic pulmonary fibrosis, interstitial lung disease, quality of life, outpatient, quality improvement, nursing

## Abstract

**Background:**

Palliative care (PC) is underutilized in the idiopathic pulmonary fibrosis (IPF) patient population, particularly in outpatient settings, despite high symptom burden and complex care needs. There is no clinician consensus for the most effective method of integrating PC into routine medical visits for this patient population, despite acknowledgement of its benefits. The purpose of this quality improvement (QI) project was to pilot an adapted nurse practitioner-led standardized PC lever tool for IPF in an outpatient clinic and evaluate the secondary PC referral rates during the implementation period.

**Design:**

The lever tool was implemented over a 3-month period. De-identified patient health information from the health system’s electronic medical record system was used to compare referrals to PC prior to and during the implementation of the lever tool.

**Results:**

The established workflow for the nurse practitioner-led implementation of the tool was feasible. There were increased PC referrals and increased PC encounters during the QI period, however the results were not statistically significant.

**Conclusions:**

The findings of this QI project add to the limited existing literature evaluating PC referral methods for individuals with IPF in an outpatient setting. Further, the development process and workflow utilized confirms the feasibility of employing the nursing workforce to support the care needs of the IPF patient population.

## Introduction

Idiopathic pulmonary fibrosis (IPF) is a subtype of chronic, progressive interstitial lung disease (ILD) of unknown etiology in which scarring gradually worsens, leading to classic symptoms of progressive dyspnea and dry cough.^1,2^ Even with the use of antifibrotic medications to slow lung scarring, there is no cure for IPF; treatment approaches focus on symptom management to improve quality of life in the face of progressive dyspnea, limited exercise tolerance, worsening lung function, and poor long-term prognosis.^3,4^ Thus, the care needs of individuals with IPF have been likened to that of patient populations with malignancies; however, access to specialized supportive care in outpatient settings is notably limited.^5^ When compared to those with lung cancer, those with ILD are less likely to receive specialized palliative care (PC) services despite the benefits.^6-8^

In their clinical practice guidelines, the American Thoracic Society/European Respiratory Society/Japanese Respiratory Society/Latin American Thoracic Association (ATS/ERS/JRS/ALAT) affirm the concurrent use of PC for the management of IPF as an adjunct to disease-directed interventions.^4^ Research has demonstrated the value of integrating PC services into care management for individuals with IPF because of its overall improvement in important care domains for patients and caregivers, such as disease education, symptom management, advance care planning (ACP), value identification, and multidisciplinary approaches to comorbidity care alignment.^9-13^ Additionally, a recent retrospective multicenter cohort study demonstrated that when used in conjunction with antifibrotics, PC may positively affect survival rates for the IPF patient population.^14^ Despite these benefits, referral rates for PC are low, not regularly assessed, happen late in disease trajectory, and vary by institution and care setting.^15-17^

A joint policy statement by ATS, American Academy of Hospice and Palliative Medicine, Hospice and Palliative Nurses Association, and Social Work Hospice and Palliative Care Network calls for incorporating the full spectrum of PC in serious respiratory illness, beginning with primary PC delivered by pulmonary clinicians and complemented by interprofessional secondary or specialist PC expertise when necessary.^8^ One significant obstacle that remains is the debate among experts regarding the best approach to integrating PC into routine medical care for IPF patients.^18^

A “levers model” was introduced for PC integration in chronic obstructive pulmonary disease (COPD).^19^ However, there is no existing comparable tool for patients with IPF. Iyer and colleagues^19^ developed a levers model to conceptualize potential triggers for early PC in COPD. In this model, four disease-related domains, including lung function, symptoms and care needs, prognosis, and severe exacerbations, have a clinically significant threshold (lever) that, when crossed, would trigger PC referral. A similar model was proposed for individuals with serious respiratory illness to describe hypothetical triggers for PC needs that exceed primary PC capability.^8^ However, it is not fully applicable to the IPF patient population, specifically in ambulatory settings given the subtleties of this disease population, such as the recommended trending of forced vital capacity (FVC) and diffusing capacity for carbon monoxide (DLCO).^4^ In this quality improvement (QI) project, we adapted and piloted a nurse practitioner-led standardized PC lever tool for IPF in an outpatient clinic and evaluated the secondary PC referral rates during the implementation period.

## Methods

### IPF-specific PC Lever Tool Development

**Figure 1.**
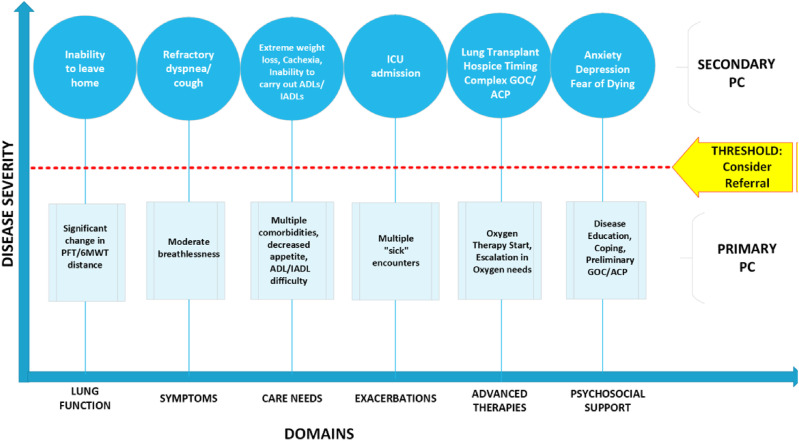
IPF-specific PC lever tool for outpatient management.

With permission, we adapted the COPD lever tool^19^ for IPF-specific use in an outpatient setting through a working group consensus. The working group included two nurse practitioners, three pulmonary attending physicians, and one PC physician. The tool was then presented to all dedicated ILD attending physicians at the Penn Medicine Harron Lung Center during a clinic-wide administration meeting for further feedback before implementation. The adapted IPF-specific lever tool (Figure 1) includes six domains (lung function, symptoms, care needs, exacerbations, advanced therapies, and psychosocial support), each with a specific threshold.

### Project Design

The project took place in the outpatient Penn Medicine Harron Lung Center in Philadelphia, PA. This is an urban academic medical setting that includes 18 specialized pulmonary clinics, including the ILD and Sarcoidosis Program from which study data were collected. This clinic comprises 13 board-certified pulmonologists, two dedicated nurse practitioners, and two dedicated registered nurses. This project was reviewed and determined to qualify as quality improvement by the University of Pennsylvania’s Institutional Review Board.

**Figure 2.**
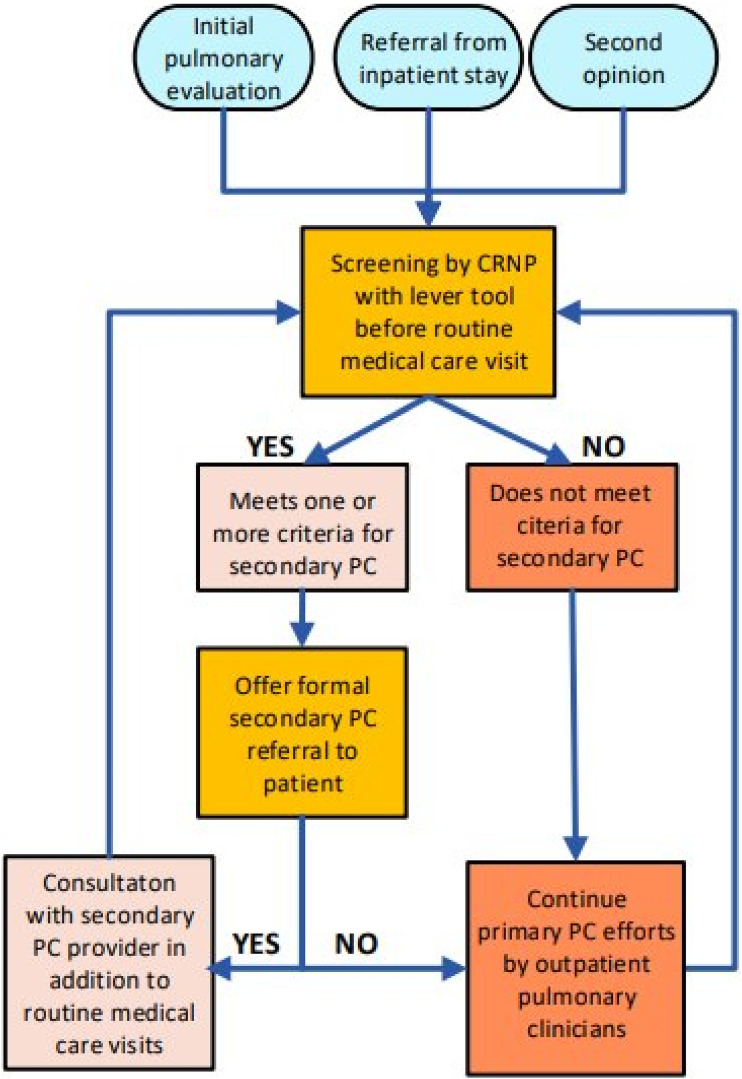
Workflow of intervention implementation in relation to clinical care.

The IPF-specific PC lever tool was implemented over a 3-month period (10/1/23 to 12/31/23). The workflow for utilization of the IPF-specific lever tool by the nurse practitioners is noted in Figure 2. Before a patient was seen in clinic for either a new patient visit, routine follow-up visit, or post-hospitalization discharge visit, a nurse practitioner screened the patient’s medical chart. The nurse practitioner screened for documentation of recent hospitalizations, calls to clinic for symptom management or care needs, and communication between providers to determine if one or more domain in the lever tool surpassed the threshold to consider a referral to secondary PC. If there was a positive screen, the nurse practitioner discussed with the attending physician the consideration of ordering a referral to secondary PC during that clinic visit. If deemed appropriate and acceptable through shared clinical decision making with the patient during the routine medical visit with the ILD care team, a formal referral was placed in the EMR for a consultation with the outpatient secondary PC team. Patients continued to be screened before each routine medical care visit to assess for changes in lever tool domains. If there was a negative screen indicating that primary PC efforts were sufficient to manage care needs, secondary PC referral was not made, and primary PC efforts continued to address care needs. Even with a negative screen, each IPF patient was reassessed before each routine medical visit to look for a change in care needs that would warrant a discussion of secondary PC consideration.

### Data Collection

We used de-identified patient health information from the health system’s electronic medical record (EMR) system, Epic, to compare referrals to PC prior to and during the implementation of the lever tool. Two quarters were assessed for comparison: quarter 1 (01/01/2023 to 03/01/2023) was the control period and quarter 4 (10/1/2023 to 12/31/2023) was the implementation period. These two time periods were chosen because of the similarity in relation to local annual increased acute respiratory care needs due to viral or bacterial infections in fall and winter months. Inclusion criteria included those with an established diagnosis of IPF per ATS guidelines^20^ as designated by their ICD-10 billing code modifier who were seen for outpatient routine medical care via in-person or telemedicine during the specified time periods.

Data were exported from the EMR with the assistance of the Penn Medicine Data Analytics Center. Data were collected in the aggregate with no patient identifiers. The base population was the distinct count of patients with the ICD-10 code for IPF (J84.112) seen for an outpatient office visit in the assigned departmental code that encompasses the location in which the majority of IPF patients are seen for outpatient management by all clinicians who see patients in that department. The same data points were extrapolated from the EMR for quarters 1 and 4 and included the following: number of PC referrals placed and number of PC-related outpatient encounters. Referrals and encounters with hospice services and home PC were included in these data points, as they are included in the continuum of PC services. Attributes of IPF patients who had PC referrals placed and were seen by PC were collected, including geographic location in relation to the medical institution, supplemental oxygen requirement, use of anti-fibrotic therapeutics, age, and gender.

### Data Analysis

Data were analyzed with IBM SPSS Statistics for Macintosh, Version 29.02.0. Data analysis utilized descriptive statistics including means, standard deviations, frequencies, and percentages to describe the sample. All analyses were based on two-tailed tests, alpha level .05. Chi-square tests were used to evaluate if there were significant differences in proportions before and during the implementation of the IPF-specific level tool in the number of outpatient referral rates of IPF patients to secondary PC services and formal encounters with secondary PC or hospice. A *P*-value less than 0.05 was considered statistically significant.

## Results

Detailed attributes and demographics of the eligible patient population analyzed from quarters 1 and 4 are included in Table 1. There were 89 eligible IPF patient care visits, including both telemedicine and in-person, in the designated outpatient department during quarter 1 and 87 during quarter 4. The mean age at the time of the clinical visit was 72 years old for both time periods. Most eligible patients were male (quarter 1: 61.8%; quarter 4: 59.8%), white (quarter 1: 87.6%; quarter 4: 86.2%) and had active orders for antifibrotic medications (quarter 1: 82%; quarter 4: 70%).

**Table 1.** Quarterly Demographics and Clinical Characteristics of Patients Before and During Quality Improvement (QI) Implementation.

	Pre-QI Period	QI Period
2023 Quarterly Intervals	Quarter 1 (1/1/2023 - 3/1/2023)	Quarter 4 (10/1/2023 - 12/31/2023)
Total Number of Patients Seen in Department 187 with IPF Diagnosis (N)	89	87
**Demographic Information**		
Patient Status, n (%)		
Alive	83 (93.2)	85 (97.7)
Deceased	6 (6.7)	2 (2.3)
Age (years), mean	72	72
Gender, n (%)		
Male	55 (61.8)	52 (59.8)
Female	34 (38.2)	25 (28.7)
Ethnicity/Race, n (%)		
White	78 (87.6)	75 (86.2)
Black	4 (4.5)	3 (3.4)
Hispanic	2 (2.2)	4 (4.6)
Other	6 (6.7)	9 (10.3)
State of Residence, n (%)		
Pennsylvania	69 (77.5)	58 (66.7)
New Jersey	24 (26.9)	21 (24.1)
Delaware	5 (5.6)	7 (8.0)
Other	1 (1.1)	1 (1.1)
**Clinical Characteristics**		
Therapy utilization, n (%)		
New/Continuing Oxygen Orders	7 (7.9)	0 (0.0)
Any Antifibrotic Therapeutic	73 (82)	61 (70.1)
Esbriet/Pirfenidone	36 (40)	24 (27.6)
Ofev/Nintedanib esylate	37 (42)	37 (42.5)

The categories reviewed to understand what effect the lever tool implementation had on PC utilization included referrals placed to hospice or PC, formal encounters with PC providers, and formal encounters with hospice providers. Table 2 provides detailed data related to secondary PC referral and utilization, as well as other outcome measures. A greater proportion of patients were referred to secondary PC or hospice during quarter 4 (8% vs quarter 1: 6%), however this difference was not statistically significant. There was also an increase in PC encounters during quarter 4 (19.5% vs quarter 1: 10.1%), but this difference was also not statistically significant. There was no effect on patients having a formal encounter with hospice providers when comparing quarter 1 to quarter 4. We reviewed ACP documentation, ED encounters, and code status documentation to further understand any additional effect that the intervention had on the patient population between quarter 1 and quarter 4. There were no statistically significant changes in any of these variables.

**Table 2.** Clinical Utilization During Quarter 1 Compared to Quarter 4 Implementation Period.

		QI Period	
Quarter	Quarter 1	Quarter 4	
Date Range	1/1/2023 – 3/1/2023	10/1/2023 -12/31/2023	*P*-value
Total Number of Patients Seen with IPF Diagnosis (N)	89	87	
Secondary PC Utilization, n (%)			
PC/Hospice Referral	5 (6.0)	7 (8.0)	*P* = 0.523
PC Encounters	9 (10.1)	17 (19.5)	*P* = 0.078
Hospice Encounters	0 (0.0)	0 (0.0)	
ACP Documented, n (%)	3 (3.4)	2 (2.3)	
ED Encounters, n (%)	29 (32.6)	24 (27.6)	
Code Status, n (%)			
Any Status Documentation	13 (14.6)	13 (14.9)	
Full Code	11 (12.4)	11 (12.6)	
DNI/DNR	2 (2.2)	2 (2.3)	
May Intubate, DNR	0 (0.0)	0 (0.0)	
Comfort Measures	0 (0.0)	0 (0.0)	

## Discussion

In this QI project, we piloted an adapted IPF-specific PC lever tool during a 3-month period in an outpatient ILD clinic and evaluated secondary PC referrals during the implementation period. The established workflow for the nurse practitioner-led implementation of the tool was feasible. Despite increased PC referrals and increased PC encounters during the QI period, the results were not statistically significant.

Similar to Zou and colleagues^15^ assertion that the referral rates to secondary PC for IPF patients are low, low referral rates were evident at our institution, even though it is a large urban academic medical center with readily available access to dedicated PC clinicians. Interestingly, referral rates during quarters 1 (6%) and 4 (8%) were lower than those of other similar academic institutions in outpatient settings, such as a previously cited rate of 13.5% of individuals with IPF over a 16-year timeframe.^15^ One reason for this could be that data were extrapolated from the EMR using ICD-10 codes alone to identify eligible patients to include in the data analysis and, thus, if coding was incorrect or not updated, data points may have been missed. Further, this QI project only included referral rates and formal encounters with secondary PC services associated with Penn Medicine; it did not include data for patients who were recommended to escalate to secondary PC services outside of this institution due to data extrapolation constraints.

Patient perspectives on PC services may have influenced results, which is noted by previous literature as being varied based on individual desire to confront different aspects of IPF trajectory and management.^21^ At this outpatient specialty clinic, testing and return visits for individuals with IPF are routinely carried out at 3- to 4-month intervals per current clinical practice guidelines.^4^ If a patient screens positive and is offered secondary PC services at an initial visit, yet experiences fear, ambivalence, or indifference, they may refuse the referral and continue primary PC services. Yet, if at subsequent visits the same patient notes that his or her symptoms or their objective testing has worsened, they may reconsider. As demonstrated in Figure 2, if a patient was rescreened at a subsequent visit and met criteria again to escalate care management to include secondary PC services and consented, it may not have been captured during the 3-month timeframe that this QI project evaluated.

## Strengths and Limitations

The findings of this QI project are valuable despite the lack of statistical significance. This project adds to the current limited literature that exists regarding different approaches to improve access to PC services for individuals with IPF. This QI project investigated a potential objective solution to addressing the need to formalize a method of evaluating PC referral needs for individuals with IPF in an outpatient setting. It used real-world clinical scenarios with extrinsic factors that evolve during routine medical care. Prior studies have identified factors that can impact integrating PC into routine medical care including patient perspectives, provider perspectives, cultural norms, systematic constraints, and health disparities.^9,22-25^ Further, specific patient attributes are known to influence PC referral; geographic location in comparison to treating facility, lung function, engagement in care, age, and comorbidity severity are cited as characteristics of IPF patients who are most often referred to PC.^15^ However, relying on such characteristics to refer IPF patients to PC may exclude individuals who fall outside of these characteristics.^15^ The lever tool adapted and implemented in this QI project identified patients in need of escalation of care outside of influences that can impact referral rates by utilizing the same approach for all individuals with the diagnosis of IPF who were seen in the clinic. Thus, one patient was not prioritized over another without a discussion of shared clinical decision making between the providers and patient. This QI project suggests that a levers model has the potential to be a valuable tool in clinical practice for managing chronic disease patient populations, like IPF, with dynamic care needs. It can be easily augmented to adjust for variables exclusive to the patient population in question based on resources available while allowing for continued shared decision making with patients and caregivers.

A further strength of this QI project is providing a trial of a specific multi-domain approach in relation to PC integration into routine clinical care for IPF patients. To address PC management thoughtfully and intentionally, this QI project modeled a multidisciplinary approach involving various clinicians, ranging from advanced practice providers to board-certified pulmonologists and PC physicians. It acknowledged efforts of pulmonary clinicians to provide primary PC efforts during routine clinical care, but also the role for expertise offered by secondary PC clinicians. The integration of PC services allows for the continual opportunity to have open discussions of topics of importance to individuals with IPF and their families, such as ACP, with knowledgeable providers.^9,26^

This QI project has several limitations. Inherent to QI project design, the results represent one department within our institution, thus the results may not be generalizable to other populations and settings. A major limitation of this QI project was the short duration of the implementation period in which the lever tool workflow was applied to routine clinical practice. This did not allow for data to be captured that reflected a reevaluation of care domains at follow-up visits for the same patient. The small sample size that this QI project included for analysis was also a limitation. Although all 13 board certified pulmonologists in this specialty clinic work with one of the two designated ILD nurse practitioners, the sample population included any patient seen in department 187 without specifying provider seen, as they often get transitioned to the ILD and Sarcoidosis Program from other subspecialties. As mentioned, de-identified patient data mined from Epic using ICD-10 codes were used, which may not have included all eligible patients if coding was not updated. Further, if a nurse practitioner was not involved in the routine medical visit for an IPF patient seen by an attending physician alone, that patient may have not been screened with the lever tool, as this was a nurse practitioner-driven initiative. The clinical role of the individual who referred a patient to secondary PC services was not looked at in this project. In the future, it would be interesting to evaluate the number of patients referred to PC by nurse practitioners alone or in tandem with attending physicians compared to attending physicians alone. Limitations in data extrapolation also did not allow for the capture of referrals to PC services and subsequent PC encounters at outside institutions. Finally, we were not able to control for the role that the impact of education on PC for clinicians had on referral rates for IPF patients to secondary PC services. During the development of the IPF-specific lever tool, information on PC services was presented to all ILD clinicians to introduce the QI project and demonstrate its use in routine medical care. However, we were not able to control for how this education impacted a clinician’s perspective on PC services, and thus, their subsequent conversations with patients during the shared decision-making process involved in this workflow.

## Implications for Future Research and Clinical Practice

This QI project was led by nurse practitioners and adds to the current evidence from Lindell and colleagues^27^ that the nursing workforce has the potential to be integral in supporting the care needs of individuals with IPF as both change agents and knowledgeable clinicians. A feasible approach to addressing PC care needs was presented using the nursing workforce to help offset previously noted concerns cited in literature regarding role confusion initiating PC discussions.^21,24,25^

The findings of this QI project demonstrate that it is possible to develop methods of formally screening individuals with IPF for an escalation in care needs that warrants a referral to secondary PC clinicians. Due to the size of this ILD clinic and number of clinicians, it had the resources to delegate this screening process to ILD subspecialty nurse practitioners who see patients both independently and in tandem with attending physicians. In the future, it would be helpful to understand if a checklist approach would be more feasible for smaller teams of providers to allow for faster screening. This approach was not used, as it was felt to be too prescriptive by the working group consensus and potentially lead to inappropriate referrals, but should be revisited. It is well documented that IPF has an unpredictable disease trajectory, which can include rapid progression, slow progression, or a stepwise decline with periods of stability after an acute decline.^4^ In the future, it would be interesting to investigate the length of time since diagnosis in relation to first consideration of secondary PC referral, as well as the impact of this on acceptance of PC services. Previous research notes that individuals with IPF and their caregivers can be beneficially impacted by PC measures that are integrated into outpatient care models.^27^ There is no consensus on the best way of doing so, but this is one proposed model that should be re-implemented during a longer period and with a larger sample size.

## Supplemental Material

Supplemental Material - The Development and Clinical Impact of an Innovative Palliative Care Lever Tool for Individuals With Interstitial Pulmonary Fibrosis: A Quality Improvement Project Supplemental Material for The Development and Clinical Impact of an Innovative Palliative Care Lever Tool for Individuals With Interstitial Pulmonary Fibrosis: A Quality Improvement Project by Kathryn Fenwick, DNP, APRN, Maryl Kreider, MD, MSCE, and Jeannette Kates in American Journal of Hospice and Palliative Medicine®
