# Supplementary material for: The Development and Clinical Impact of an Innovative Palliative Care Lever Tool for Individuals With Idiopathic Pulmonary Fibrosis: A Quality Improvement Project
Source: Am J Hosp Palliat Care. 2024 Nov 29;42(11):1161–8. doi: 10.1177/10499091241304443 (PMC12433531; doi:10.1177/10499091241304443)
Supplement: Supplemental Material - The Development and Clinical Impact of an Innovative Palliative Care Lever Tool for Individuals With Interstitial Pulmonary Fibrosis: A Quality Improvement Project [file sj-pdf-1-ajh-10.1177_10499091241304443.pdf]

Appendix A

Copyright Clearance for Use of Tool by Iyers and colleagues (2022)

ELSEVIER LICENSE
TERMS AND CONDITIONS

Jul 26, 2024

This Agreement between Kathryn Elizabeth Fenwick ("You") and Elsevier ("Elsevier") consists of your license details and the terms and conditions provided by Elsevier and Copyright Clearance Center.

License Number	5834480908568
License date	Jul 22, 2024
Licensed Content Publisher	Elsevier
Licensed Content Publication	CHEST
Licensed Content Title	The Role of Palliative Care in COPD
Licensed Content Author	Anand S. Iyer,Donald R. Sullivan,Kathleen O. Lindell,Lynn F. Reinke
Licensed Content Date	May 1, 2022
Licensed Content Volume	161
Licensed Content Issue	5
Licensed Content Pages	13
Start Page	1250
End Page	1262
Type of Use	reuse in a journal/magazine
Requestor type	academic/educational institute
Portion	figures/tables/illustrations
Number of figures/tables/illustrations	1
Format	both print and electronic
Are you the author of this Elsevier article?	Yes
Will you be translating?	No
Title of new article	The Development and Clinical Utility of an Innovative Palliative Care Lever Tool for Individuals with Idiopathic Pulmonary Fibrosis
Lead author	Kathryn Elizabeth Fenwick, DNP, FNP-BC
Title of targeted journal	American Journal of Hospice and Palliative Medicine

Publisher	SAGE Publications
Expected publication date	Dec 2024
Order reference number	4174
Portions	Figure 3. Diagram showing the levers model for palliative care integration in COPD.
The Requesting Person / Organization to Appear on the License	Kathryn Elizabeth Fenwick
Requestor Location	Jefferson College of Nursing<br>743 South 20th Street<br>APT 3<br><br>PHILADELPHIA, PA 19146<br>United States<br>Attn: Jefferson College of Nursing
Publisher Tax ID	98-0397604
Total	0.00 USD
